# Supplementary material for: DisA Limits RecG Activities at Stalled or Reversed Replication Forks
Source: Cells. 2021 May 31;10(6):1357. doi: 10.3390/cells10061357 (PMC8227628; doi:10.3390/cells10061357)
Supplement: Supplementary file 1 [file cells-10-01357-s001.zip › cells-1210215-supplementary.pdf]

cells

MDPI

Supplementary material

Article

DisA Limits RecG Activities at Stalled or Reversed Replication Forks

Rubén Torres, Carolina Gándara †, Begoña Carrasco, Ignacio Baquedano ‡, Silvia Ayora and Juan C. Alonso *

Department of Microbial Biotechnology, Centro Nacional de Biotecnología, CNB-CSIC, 28049 Madrid, Spain; rtorres@cnb.csic.es (R.T.); carolina.gandara@newcellsbiotech.co.uk (C.G.); bcarrasc@cnb.csic.es (B.C.); ignacio.baquedano@ua.es (I.B.); sayora@cnb.csic.es (S.A.)

† Present address: Newcells Biotech Ltd., Newcastle Helix, Newcastle-upon-Tyne NE4 5BX, UK

‡ Present address: Departamento de Fisiología, Genética y Microbiología. Universidad de Alicante. 03690 San Vicente del Raspei, Alicante, Spain

* Correspondence: jcalonso@cnb.csic.es

Table S1. Oligonucleotides used

Name	Sequence 5´→3´polarity
J3-1	CGCAAGCGACAGGAACCTCGAGAAGCTTCCGGTAGCAGCCTGAGCGGTGGTTGAATTCCTC GAGGTTCCTGTCGCTTGCG
J3-2	CGCAAGCGACAGGAACCTCGAGGAATTCAACCACCGCTCAACTCAACTGCAGTCTAGACTC GAGGTTCCTGTCGCTTGCG
J3-3	CGCAAGCGACAGGAACCTCGAGTCTAGACTGCAGTTGAGTCCTTGCTAGGACGGATCCCTC GAGGTTCCTGTCGCTTGCG
J3-4	CGCAAGCGACAGGAACCTCGAGGGATCCGTCCTAGCAAGGGGCTGCTACCGGAAGCTTCTC GAGGTTCCTGTCGCTTGCG
J3-2-110	CGCAAGCGACAGGAACCTCGAGGAATTCAACCACCGCTCAACTCAACTGCAGTCTAGACTC GAGGTTCCTGTCGCTTGCGAAGTCTTTCCGGCATCGATCGTAGCTATTT
J3-2-110-5	AAGTCTTTCCGGCATCGATCGTAGCTATTTCGCAAGCGACAGGAACCTCGAGGAATTCAAC CACCGCTCAACTCAACTGCAGTCTAGACTCGAGGTTCCTGTCGCTTGCG
J170	AGACGCTGCCGAATTCTGGCTTGGATCTGATGCTGTCTAGAGGCCTCCACTATGAAATCG
J173	AGCTCATAGATCGATAGTCTCTAGACAGCATCAGATCCAAGCCAGAATTCGGCAGCGTCT
J345	GCGATTTCATAGTGGAGGCCTCTAGACAGCACGCCGTTGAATGGGCGGATGCTAATTACTAT CTC
J346	GAGATAGTAATTAGCATCCGCCCATTCAACGGCGTGCTGTCTAGAGACTATCGATCTATGAG CTCTGCAGC
171	CGATTTCATAGTGGAGGCCTCTAGACAGCA
172	TGCTGTCTAGAGACTATCGATCTATGAGCT
171-15	CGATTTCATAGTGGA
172-15	ATCGATCTATGAGCT

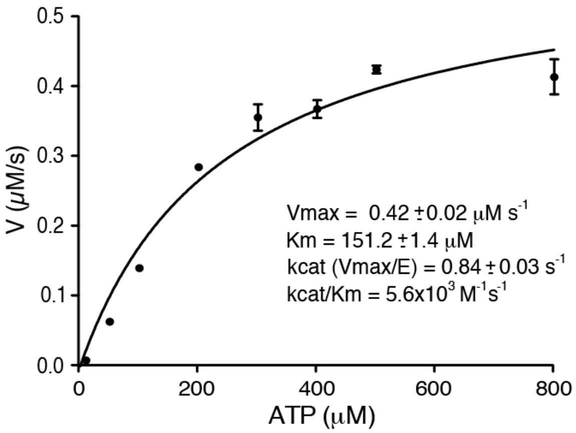

Figure S1. DisA DAC activity at different ATP concentrations**.** DisA (25 nM) was incubated in buffer C containing 10 mM $MgCl_2$ and increasing ATP concentrations (at a ratio of 1:2000, 1:4000 or 1:8000 [α^{32}P]-ATP:ATP) for 30 min at 37 °C. Samples were separated by TLC. Spots corresponding to ATP and c-di-AMP were quantified using the ImageJ (NIH) program, and data, which are plotted in a Michaelis-Menten kinetic curve, are the mean ± SD of >3 independent experiments. The obtained kinetic parameters for DisA-mediated c-di-AMP synthesis are shown.

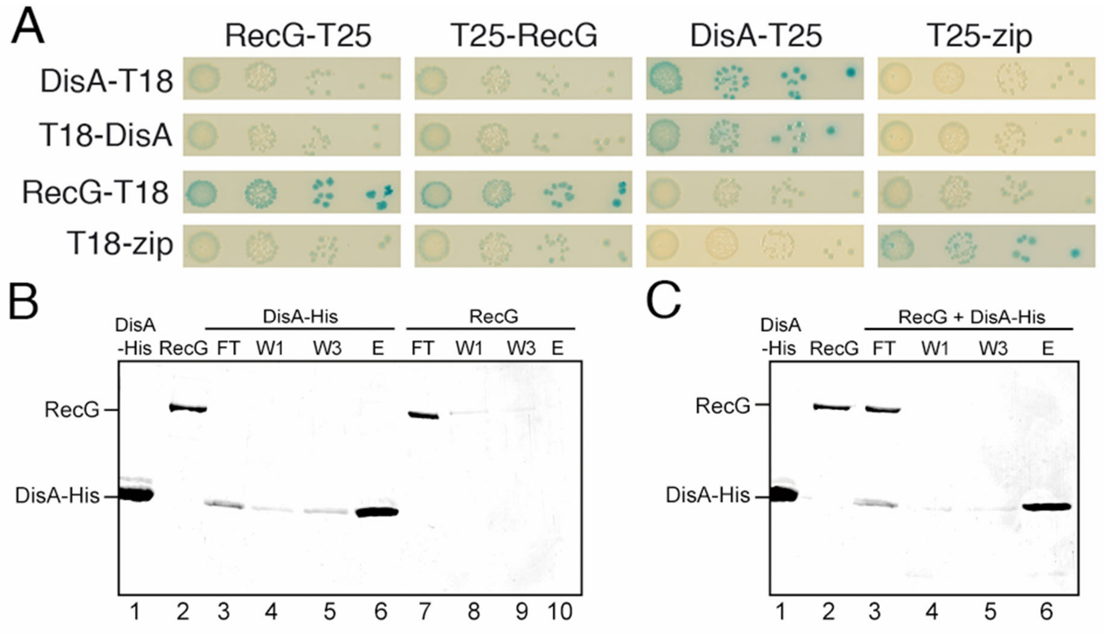

Figure 2. DisA does not form a stable complex with RecG. (**A**) Bacterial two-hybrid interaction assays were done by co-transforming a pair of plasmids expressing DisA, RecG or the Zip control fused at the N- or C-terminus, to either the T18 or T25 domain of the adenylate cyclase. Dilutions of co-transformants were incubated on plates supplemented with IPTG and X-Gal. A positive interaction (appearance of blue color) is considered when the pair of fusion proteins reconstitutes the adenylate cyclase to generate cAMP, which then activates the cAMP-bound catabolite activator protein to induce the expression of β-galactosidase. Different dilutions were spotted onto LB plates supplemented with ampicillin, kanamycin, streptomycin, 0.5 mM IPTG and 10% X-Gal. The plates were then incubated at 25 °C for 3–4 days. Each co-transformation was performed at least in triplicate and a representative result is shown. (**B**) His-tagged DisA (1 µg) or RecG (0.5 µg) was loaded onto a 50 µl Ni^{2+} matrix in buffer B, and the FT was collected. The Ni^{2+} matrix was washed three times with four column volumes of buffer B containing 20 mM imidazole (W), and the bound proteins were finally eluted with Buffer B containing 0.4 M imidazole. (**C**) His-tagged DisA was first incubated with RecG in buffer B (5 min, 37ºC), and then the mixture was loaded onto a 50 µl Ni^{2+} matrix. The FT was collected, and three washes at 20 mM imidazole were performed. Finally, bound His-tagged DisA was eluted with Buffer B containing 0.4 M imidazole. The experiment was repeated three times with similar results, and a representative gel is shown here.

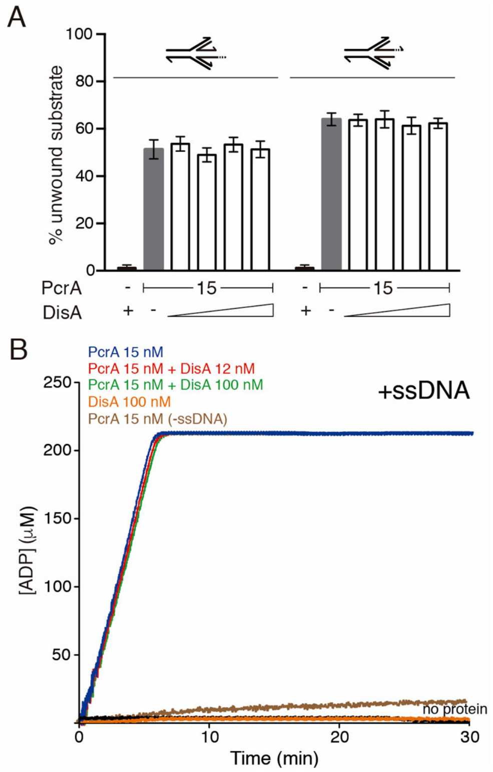

Figure S3. The activities of the PcrA helicase are not regulated by DisA. (**A**) PcrA-mediated helicase assays with a 5′-tail HJ DNA (it mimics a regressed fork with the nascent lagging-strand 30-nt longer than the nascent lagging-strand) or a 3′-tail HJ DNA (regressed fork with the nascent leading-strand 30-nt longer than the nascent lagging-strand). The DNA was incubated with PcrA (15 nM) and increasing concentrations of DisA (12-100 nM). Reactions were done in buffer B containing 10 mM $MgCl_2$ and 2 mM ATP (15 min, 30 °C), and after deproteinization the substrate and products were separated by 6% PAGE and visualized by phosphor imaging. The quantification values of unwound DNA and the SD of >3 independent experiments are documented. Abbreviations: - and +, absence and presence of the indicated protein. (**B**) PcrA-mediated ATP hydrolysis in the presence of DisA. Reactions had 15 nM PcrA, the indicated DisA concentrations and circular 3,199-nt ssDNA (10 μM in nt) in buffer D containing the ATP regeneration system. Reactions were started by addition of ATP (5 mM), and the ATPase activity was measured (30 min at 37 °C). All reactions were repeated three or more times with similar results. A representative graph is shown here, and quantifications of the ATP hydrolysis rates are shown in the main text as the mean ± SD of >3 independent experiments.
